# Supplementary material for: Loss of years of healthy life due to road incidents of motorcyclists in the city of Medellin, 2012 to 2015
Source: PLoS One. 2021 Aug 27;16(8):e0256758. doi: 10.1371/journal.pone.0256758 (PMC8396781; doi:10.1371/journal.pone.0256758)
Supplement: S1 Table — (DOCX) [file pone.0256758.s001.docx]

**S1 Table. Nature of lesions and assigned ICD10 codes**

| **ID** | **Nature of injury** | **ICD-10** |
| --- | --- | --- |
|  |  |  |
| **1** | *Amputation of finger(s), excluding thumb | S68.1, S68.2, S68.6 |
| **2** | *Amputation of thumb (long term) | S68.0 |
| **3** | Amputation of one upper limb (long term, with treatment) | S48.9, S58.1, S68.3, S68.9, T05.0, T05.2, T11.6 |
| **3** | *Amputation of one upper limb (long term, with treatment) | S48.9, S58.1, S68.3, S68.9, T05.0, T05.2, T11.6 |
| **4** | Amputation of both upper limbs (long term, with treatment) | S68.4 |
| **4** | *Amputation of both upper limbs (long term, with treatment) | S68.4 |
| **5** | *Amputation of toe(s) | S98.1, S98.2 |
| **6** | Amputation of one lower limb (long term, with treatment) | S78.0, S78.1, S78.9, S88.9, S98.0, S98.3, S98.4, S98.9, T05.3, T05.4, T05.6, T13.6 |
| **6** | *Amputation of one lower limb (long term, with treatment) | S78.0, S78.1, S78.9, S88.9, S98.0, S98.3, S98.4, S98.9, T05.3, T05.4, T05.6, T13.6 |
| **7** | Amputation of both lower limbs (long term, with treatment) | - |
| **7** | *Amputation of both lower limbs (long term, with treatment) | - |
| **8** | *Burns, <20% total burned surface area without lower airway burns (short term, with or without treatment) | T20.0, T20.1, T20.2, T20.4, T20.6, T20.7, T21.0, T21.1, T21.2, T21.4, T21.7, T23.1, T24.5, T25.0, T25.1, T25.2, T25.3, T25.4, T25.5, T25.6, T25.7, T28.3, T28.4, T31.0, T31.1 |
| **9** | *Burns, <20% total burned surface area or <10% total burned surface area if head/neck or hands/wrist involved (long term, with or without treatment) | - |
| **10** | *Burns, >20% total burned surface area (short term, with or without treatment) | T29.6, T31.4, T31.5, T31.6, T31.7, T31.8, T31.9, T32.2, T32.4, T32.9 |
| **10** | Burns, >20% total burned surface area (short term, with or without treatment) | T29.6, T31.4, T31.6, T31.8, T31.9, T32.2, T32.4, T32.9 |
| **10** | Burns, >20% total burned surface area (short term, with or without treatment) | T29.6, T31.4, T31.6, T31.8, T31.9, T32.2, T32.4, T32.9 |
| **11** | *Lower airway burns (with or without treatment) | T27.3 |
| **12** | *Crush injury (short or long term, with or without treatment) | S07.0, S07.1, S07.8, S17.0, S17.8, S17.9, S38.0, S67.0, S67.1, S67.3, S67.9, S77.1, S77.2, S87.8, S97.1, S97.8, T04, T14.7, T92.6, T93.6 |
| **13** | *Dislocation of hip (long term, with or without treatment) | S73.0 |
| **14** | *Dislocation of knee (long term, with or without treatment) | S83.0 |
| **15** | *Dislocation of shoulder (long term, with or without treatment) | S43.0, S43.1, S43.2, S43.3 |
| **16** | *Other injuries of muscle and tendon (includes sprains, strains and dislocations other than shoulder, knee, hip) | S03.0, S03.1, S03.2, S03.3, S03.8, S03.9, S13.0, S13.1, S13.2, S13.3, S13.4, S13.5, S13.6, S16.1, S16.2, S16.8, S16.9, S23.0, S23.1, S23.2, S23.3, S23.4, S23.5, S33.3, S43.4, S43.9, S46.1, S46.2, S46.8, S46.9, S53.0, S53.1, S53.4, S66.2, S66.3, S73.1, S76.0, S76.1, S76.2, S76.3, S76.8, S76.9, S86.0, S86.1, S86.2, S86.3, S86.8, S93.0, S93.1, S93.3, S93.4, S93.5, S93.6, S96.0, S96.1, S96.2, S96.9, S99.9 |
| **17** | *Drowning and nonfatal submersion (short or long term, with or without treatment) | - |
| **18** | *Fracture of clavicle, scapula or humerus (short or long term, with or without treatment) | S49.0, S49.1 |
| **19** | *Fracture of face bone (short or long term, with or without treatment) | S02.2, S02.3, S02.4, S02.5, S02.6, S02.7 |
| **20** | *Fracture of foot bones (short term, with or without treatment)_ except ankle | S92.3, S92.4, S92.5, S92.7, S92.9, S99.7 |
| **20** | Fracture of foot bones (short term, with or without treatment)_ except ankle | S92.3, S92.4, S92.5, S92.7, S92.9, S99.7 |
| **21** | *Fracture of hand (short term, with or without treatment) | S62.8 |
| **21** | Fracture of hand (short term, with or without treatment) | S62.8 |
| **22** | *Fracture of neck of femur /fracture of hip (short term, with or without treatment) | S72.0, S72.1, S72.2 |
| **22** | Fracture of neck of femur /fracture of hip (short term, with or without treatment) | S72.0, S72.1, S72.2 |
| **22** | Fracture of neck of femur /fracture of hip (short term, with or without treatment) | S72.0, S72.1, S72.2 |
| **23** | *Fracture of femur, other than femoral neck (short term, with or without treatment) | S79.1, T93.1 |
| **23** | Fracture of femur, other than femoral neck (short term, with or without treatment) | S79.1, T93.1 |
| **24** | *Fracture of patella, tibia or fibula or ankle (short term, with or without treatment) | S82.0, S82.1, S82.2, S82.3, S82.4, S82.5, S82.6, S82.7, S82.8, S82.9, S89.0, S89.1, S89.2, S89.3, T12 |
| **24** | Fracture of patella, tibia or fibula or ankle (short term, with or without treatment) | S82.0, S82.1, S82.2, S82.3, S82.4, S82.5, S82.6, S82.7, S82.8, S82.9, S89.0, S89.1, S89.2, S89.3, T12 |
| **25** | *Fracture of pelvis (short term) | S32.5, S32.1, S32.8, T91.2 |
| **25** | Fracture of pelvis (short term) | S32.5, S32.1, S32.8, T91.2 |
| **26** | *Fracture of radius or ulna (short term, with or without treatment) | S52.3, S52.4, S52.5, S52.6, S52.7, S59.0, S59.1, S59.2, T10, T92.1 |
| **26** | Fracture of radius or ulna (short term, with or without treatment) | S52.3, S52.4, S52.5, S52.6, S52.7, S59.0, S59.1, S59.2, T10, T92.1 |
| **27** | *Fracture of skull (short or long term, with or without treatment) | S02.0, S02.1, S02.8, S02.9 |
| **28** | *Fracture of sternum and/or fracture of one or two ribs (short term, with or without treatment) | S22.2, S22.3, S22.4, S22.8, S22.9 |
| **29** | *Fracture of vertebral column (short or long term, with or without treatment) | S12.0, S12.5, S12.6, S22.0, S22.1 , S32.0, S32.7, T91.1 |
| **30** | *Fractures, treated (long term) | - |
| **31** | *Injured nerves (short term) | S04.0, S04.1, S04.2, S04.3, S04.4, S04.5, S04.6, S04.7, S04.8, S04.9, S14.2, S14.3, S14.4, S14.5, S14.6, S14.8, S34.8, S34.9, S44.5, S54.0, S54.1, S54.2, S54.3, S64.4, S64.8, S64.9, S74.0, S74.1, S74.2, S74.9, S94.0, S94.1, T13.3, T90.3 |
| **31** | Injured nerves (short term) | S04.0, S04.1, S04.2, S04.3, S04.4, S04.5, S04.6, S04.7, S04.8, S04.9, S14.2, S14.3, S14.4, S14.5, S14.6, S14.8, S34.8, S34.9, S44.5, S54.0, S54.1, S54.2, S54.3, S64.4, S64.8, S64.9, S74.0, S74.1, S74.2, S74.9, S94.0, S94.1, T13.3, T90.3 |
| **32** | *Injury to eyes (short term) | S01.1, S05.0, S05.1, S05.2, S05.3, S05.4, S05.5, S05.6, S05.7, S05.8, S05.9, T15.0, T15.1, T15.8, T26.4, T26.5, T26.6, T26.8, T90.4 |
| **33** | *Concussion | S06.0 |
| **34** | *Severe traumatic brain injury, short term (with or without treatment) | S06.1, S06.2, S06.3, S06.4, S06.5, S06.6, S06.7, S06.8, S06.9, T90.2 |
| **35** | *Traumatic brain injury, long-term consequences, minor (with or without treatment) | G44.3, S06.0 |
| **36** | *Traumatic brain injury, long-term consequences, moderate (with or without treatment) | S06.1, S06.2, S06.3, S06.4, S06.5, S06.6, S06.7, S06.8, S06.9, T90.2 |
| **37** | *Traumatic brain injury, long-term consequences, severe (with or without treatment) | S06.1, S06.2, S06.3, S06.4, S06.5, S06.6, S06.7, S06.8, S06.9, T90.2 |
| **38** | *Open wound (short term, with or without treatment) | S01.0, S01.2, S01.3, S01.4, S01.5, S01.7, S01.8, S01.9, S08.0, S08.1, S08.8, S09.0, S09.1, S09.2, S09.3, S10.7, S11.1, S11.8, S11.9, S15.0, S15.1, S15.2, S15.3, S15.7, S15.8, S15.9, S21.0, S21.1, S21.2, S21.3, S21.4, S21.7, S21.8, S21.9, S31.8, S41.0, S41.1, S45.1, S45.3, S51.0, S51.8, S55.0, S55.1, S55.8, S55.9, S65.0, S65.3, S65.4, S65.5, S65.7, S65.8, S65.9, S71.0, S71.1, S71.7, S75.0, S75.1, S75.2, S75.8, S75.9, S81.0, S81.7, S81.8, S81.9, S85.1, S85.2, S85.3, S85.4, S85.5, S85.8, S85.9, S95.0, S95.2, S95.8, T90.1, T93.0 |
| **39** | *Poisoning (short term with or without treatment) | T36.9, T38.8, T38.9, T39.0, T39.3, T39.8, T39.9, T40.3, T40.4, T40.5, T40.6, T40.9, T41.2, T41.4, T42.7, T43.0, T43.2, T43.4, T43.5, T43.6, T43.9, T44.6, T44.9, T45.5, T45.6, T45.7, T45.8, T45.9, T46.0, T46.1, T46.2, T46.3, T46.4, T46.5, T46.6, T46.7, T46.8, T46.9, T47.0, T47.1, T47.2, T47.3, T47.4, T47.5, T47.6, T47.7, T47.8, T47.9, T48.0, T48.1, T48.2, T48.3, T48.4, T48.5, T48.6, T48.7, T48.9, T49.0, T49.1, T49.2, T49.3, T49.4, T49.5, T49.6, T49.7, T49.8, T49.9, T50.0, T50.3, T50.4, T50.8, T50.9, T51.0, T51.1, T51.2, T52.4, T53.5, T54.9, T56.4, T56.8, T57.9, T58.1, T58.2, T58.8, T58.9, T59.0, T59.1, T59.2, T59.3, T59.4, T59.5, T59.6, T59.9, T60.9, T61.1, T61.7, T62.9, T63.8, T65.2, T65.8, T65.9 |
| **40** | Severe chest injury (long term, with or without treatment) | S11.0, S11.2, S25.0, S25.1, S25.2, S25.3, S25.4, S25.5, S25.7, S25.8, S25.9, S26.0, S26.1, S27.3, S27.4, S27.8, S28.2, T91.4 |
| **40** | *Severe chest injury (long term, with or without treatment) | S11.0, S11.2, S25.0, S25.1, S25.2, S25.3, S25.4, S25.5, S25.7, S25.8, S25.9, S26.0, S26.1, S27.3, S27.4, S27.8, S28.2, T91.4 |
| **41** | Spinal cord lesion below neck level (treated) | S24.0, S24.1, S34.1 |
| **41** | *Spinal cord lesion below neck level (treated) | S24.0, S24.1, S34.1 |
| **42** | Spinal cord lesion at neck level (treated) | S14.1, T91.3 |
| **42** | *Spinal cord lesion at neck level (treated) | S14.1, T91.3 |
| **43** | *Internal hemorrhage in abdomen and pelvis | S35.1, S35.2, S35.3, S35.4, S35.5, S35.9, S36.0, S36.1, S36.2, S36.3, S36.4, S36.5, S36.6, S36.8, S37.0, S37.2, S37.3, S37.4, S37.5, S37.8, S37.9, T79.6 |
| **44** | *Contusion in any part of the body | S20.0, S30.2, S40.2, S50.0, S60.2, S60.8, S70.0, S80.0, S80.1, S80.2, S80.7, S90.0, S90.2 |
| **45** | *Superficial injury of any part of the body | S00.0, S00.1, S00.2, S00.3, S00.4, S00.5, S00.8, S00.9, S10.0, S10.1, S10.8, S10.9, S20.1, S20.3, S20.9, S30.8, S40.2, S40.7, S40.8, S40.9, S50.3, S50.7, S50.8, S70.2, S70.3, S80.8, S80.9, S90.4, S90.5, S90.8, S90.9, T00.8, T00.9, T90.0 |
| **46** | Multiple fractures, dislocations, crashes, wounds , sprains, and strains | T02.7, T04.7, T06.3 |

**Note:** * Nature of injury considered in this study.

**Source:** Annex to The global burden of injury: incidence, mortality, disability-adjusted life year estimates and time trends from the Global Burden of Disease Study 2013. WHO methods and data sources for global burden of disease estimates 2000-2015*.*
