# Supplementary material for: Loss of years of healthy life due to road incidents of motorcyclists in the city of Medellin, 2012 to 2015
Source: PLoS One. 2021 Aug 27;16(8):e0256758. doi: 10.1371/journal.pone.0256758 (PMC8396781; doi:10.1371/journal.pone.0256758)
Supplement: S2 Table — (DOCX) [file pone.0256758.s002.docx]

**S2 Table. Disability weights by nature of injury according to GBD**

| **ID** | **Nature of injury** | **Disability weight** | | |
| --- | --- | --- | --- | --- |
|  |  | **GBD 2013** | | |
|  |  | Estimate | 95% CI | |
| **1** | *Amputation of finger(s), excluding thumb | 0.005 | 0.002 | 0.01 |
| **2** | *Amputation of thumb (long term) | 0.011 | 0.005 | 0.021 |
| **3** | *Amputation of one upper limb (long term, with treatment) | 0.118 | 0.079 | 0.167 |
| **4** | *Amputation of both upper limbs (long term, with treatment) | 0.383 | 0.251 | 0.525 |
| **5** | *Amputation of toe(s) | 0.006 | 0.002 | 0.012 |
| **6** | *Amputation of one lower limb (long term, with treatment) | 0.173 | 0.118 | 0.24 |
| **7** | *Amputation of both lower limbs (long term, with treatment) | 0.443 | 0.297 | 0.589 |
| **8** | *Burns, <20% total burned surface area without lower airway burns (short term, with or without treatment) | 0.141 | 0.094 | 0.196 |
| **9** | *Burns, <20% total burned surface area or <10% total burned surface area if head/neck or hands/wrist involved (long term, with or without treatment) | 0.016 | 0.008 | 0.028 |
| **10** | *Burns, >20% total burned surface area (short term, with or without treatment) | 0.314 | 0.211 | 0.441 |
| **11** | *Lower airway burns (with or without treatment) | 0.376 | 0.24 | 0.524 |
| **12** | *Crush injury (short or long term, with or without treatment) | 0.132 | 0.089 | 0.189 |
| **13** | *Dislocation of hip (long term, with or without treatment) | 0.016 | 0.008 | 0.028 |
| **14** | *Dislocation of knee (long term, with or without treatment) | 0.113 | 0.075 | 0.16 |
| **15** | *Dislocation of shoulder (long term, with or without treatment) | 0.062 | 0.041 | 0.088 |
| **16** | *Other injuries of muscle and tendon (includes sprains, strains and dislocations other than shoulder, knee, hip) | 0.008 | 0.003 | 0.015 |
| **17** | *Drowning and nonfatal submersion (short or long term, with or without treatment) | 0.247 | 0.164 | 0.341 |
| **18** | *Fracture of clavicle, scapula or humerus (short or long term, with or without treatment) | 0.035 | 0.021 | 0.053 |
| **19** | *Fracture of face bone (short or long term, with or without treatment) | 0.067 | 0.044 | 0.097 |
| **20** | *Fracture of foot bones (short term, with or without treatment)_ except ankle | 0.026 | 0.015 | 0.043 |
| **21** | *Fracture of hand (short term, with or without treatment) | 0.01 | 0.005 | 0.019 |
| **22** | *Fracture of neck of femur /fracture of hip (short term, with or without treatment) | 0.258 | 0.172 | 0.356 |
| **23** | *Fracture of femur, other than femoral neck (short term, with or without treatment) | 0.111 | 0.074 | 0.156 |
| **24** | *Fracture of patella, tibia or fibula or ankle (short term, with or without treatment) | 0.05 | 0.032 | 0.075 |
| **25** | *Fracture of pelvis (short term) | 0.279 | 0.188 | 0.384 |
| **26** | *Fracture of radius or ulna (short term, with or without treatment) | 0.028 | 0.016 | 0.046 |
| **27** | *Fracture of skull (short or long term, with or without treatment) | 0.071 | 0.048 | 0.1 |
| **28** | *Fracture of sternum and/or fracture of one or two ribs (short term, with or without treatment) | 0.103 | 0.068 | 0.145 |
| **29** | *Fracture of vertebral column (short or long term, with or without treatment) | 0.111 | 0.075 | 0.156 |
| **30** | *Fractures, treated (long term) | 0.005 | 0.002 | 0.01 |
| **31** | *Injured nerves (short term) | 0.1 | 0.067 | 0.14 |
| **32** | *Injury to eyes (short term) | 0.054 | 0.035 | 0.081 |
| **33** | *Concussion | 0.11 | 0.074 | 0.158 |
| **34** | *Severe traumatic brain injury, short term (with or without treatment) | 0.214 | 0.141 | 0.297 |
| **35** | *Traumatic brain injury, long-term consequences, minor (with or without treatment) | 0.094 | 0.063 | 0.133 |
| **36** | *Traumatic brain injury, long-term consequences, moderate (with or without treatment) | 0.231 | 0.156 | 0.324 |
| **37** | *Traumatic brain injury, long-term consequences, severe (with or without treatment) | 0.637 | 0.462 | 0.789 |
| **38** | *Open wound (short term, with or without treatment) | 0.006 | 0.002 | 0.012 |
| **39** | *Poisoning (short term with or without treatment) | 0.163 | 0.109 | 0.227 |
| **40** | *Severe chest injury (short term, with or without treatment) | 0.369 | 0.248 | 0.501 |
| **41** | *Spinal cord lesion below neck level (without treatment) | 0.623 | 0.434 | 0.777 |
| **42** | *Spinal cord lesion at neck level (without treatment) | 0.732 | 0.544 | 0.871 |
| **43** | *Internal hemorrhage in abdomen and pelvis | 0.279** | 0.188 | 0.384 |
| **44** | *Contusion in any part of the body | 0.008*** | 0.003 | 0.015 |
| **45** | *Superficial injury of any part of the body | 0.008*** | 0.003 | 0.015 |
| **46** | *Multiple fractures, dislocations, crashes, wounds , sprains, and strains | 0.062**** |  |  |

**Note:** * Nature of injury considered in this study. ** It is left the same as nature 25, because "Internal bleeding includes severe injuries that can cause rapid heartbeat, cold pale skin, sweating and even loss of consciousness. They can be caused by crushing, trauma to the abdomen or chest with injuries organs and blood vessels or fractures of the pelvis or lower limbs" (www.ecured.cu/Hemorragia_interna). *** Left unchanged 16: Other muscle and tendon injuries (includes sprains, strains, and dislocations other than shoulder, knee, hip). **** Median fractures, dislocations, sprains, and strains were pulled.

**Fuente:** Annex to The global burden of injury: incidence, mortality, disability-adjusted life year estimates and time trends from the Global Burden of Disease Study 2013. WHO methods and data sources for global burden of disease estimates 2000-2015.
