# Supplementary material for: Loss of years of healthy life due to road incidents of motorcyclists in the city of Medellin, 2012 to 2015
Source: PLoS One. 2021 Aug 27;16(8):e0256758. doi: 10.1371/journal.pone.0256758 (PMC8396781; doi:10.1371/journal.pone.0256758)
Supplement: S3 Table — (DOCX) [file pone.0256758.s003.docx]

**S3 Table. Number of deaths, on-site injuries, injuries attended, YLL, YLD and DALY for motorcyclist road incidents per year, according to sex and age groups, Medellín, 2012-2015.**

| **Year** | **Age group** | **Men** | | | | | | | **Women** | | | | | | | **Total** | | | | | | |
| --- | --- | --- | --- | --- | --- | --- | --- | --- | --- | --- | --- | --- | --- | --- | --- | --- | --- | --- | --- | --- | --- | --- |
|  |  | **Population** | **M*** | **L1** | **L2** | **YLLS** | **YLDS** | **DALYS** | **Population** | **M*** | **L1** | **L2** | **YLLS** | **YLDS** | **DALYS** | **Population** | **M*** | **L1** | **L2** | **YLLS** | **YLDS** | **DALYS** |
| **2012** | **1-4** | 74.674 | 1 | 24 | - | 92 |  | 92 | 71.535 | - | 22 | - | 0 |  | 0 | 146.209 | 1 | 46 | - | 92 | 0 | 92 |
|  | **5-9** | 76.448 | - | 55 | - | 0 |  | 0 | 72.645 | - | 22 | 1 | 0 | 0 | 0 | 149.093 | - | 77 | 1 | 0 | 0 | 0 |
|  | **10-14** | 82.638 | - | 133 | 6 | 0 | 1 | 1 | 79.711 | 1 | 103 | 5 | 79 | 2 | 81 | 162.349 | 1 | 236 | 11 | 79 | 2 | 82 |
|  | **15-19** | 93.581 | 10 | 1.726 | 586 | 755 | 762 | 1517 | 90.024 | - | 573 | 287 | 0 | 184 | 184 | 183.605 | 10 | 2.300 | 873 | 755 | 946 | 1701 |
|  | **20-24** | 103.049 | 28 | 4.085 | 1.398 | 1978 | 1836 | 3814 | 100.118 | 6 | 1.212 | 555 | 428 | 555 | 983 | 203.167 | 34 | 5.297 | 1.953 | 2406 | 2391 | 4797 |
|  | **25-29** | 96.342 | 16 | 3.193 | 1.262 | 1053 | 1402 | 2455 | 100.109 | - | 966 | 558 | 0 | 444 | 444 | 196.451 | 16 | 4.159 | 1.820 | 1053 | 1846 | 2899 |
|  | **30-34** | 81.014 | 18 | 2.169 | 881 | 1089 | 943 | 2032 | 91.403 | 3 | 669 | 414 | 183 | 280 | 463 | 172.417 | 21 | 2.838 | 1.295 | 1272 | 1223 | 2495 |
|  | **35-39** | 68.120 | 7 | 1.176 | 539 | 391 | 585 | 976 | 81.114 | 3 | 343 | 260 | 167 | 102 | 269 | 149.234 | 10 | 1.519 | 799 | 558 | 687 | 1244 |
|  | **40-44** | 77.118 | 5 | 1.228 | 458 | 253 | 366 | 618 | 92.012 | - | 520 | 196 | 0 | 36 | 36 | 169.130 | 5 | 1.748 | 654 | 253 | 401 | 654 |
|  | **45-49** | 88.835 | 3 | 555 | 363 | 136 | 133 | 269 | 107.450 | 1 | 148 | 194 | 44 | 60 | 104 | 196.285 | 4 | 703 | 557 | 180 | 193 | 373 |
|  | **50-54** | 81.296 | 2 | 302 | 209 | 84 | 34 | 117 | 101.827 | - | 89 | 148 | 0 | 35 | 35 | 183.123 | 2 | 391 | 357 | 84 | 68 | 152 |
|  | **55-59** | 64.597 | 1 | 156 | 156 | 71 | 125 | 196 | 81.828 | - | 40 | 126 | 0 | 16 | 16 | 146.425 | 1 | 196 | 282 | 71 | 142 | 212 |
|  | **60-64** | 49.753 | - | 74 | 123 | 0 | 103 | 103 | 64.046 | - | 20 | 77 | 0 | 9 | 9 | 113.799 | - | 93 | 200 | 0 | 112 | 112 |
|  | **65 y más** | 89.130 | - | 72 | 205 | 0 | 247 | 247 | 132.594 | - | 2 | 182 | 0 | 98 | 98 | 221.724 | - | 85 | 387 | 0 | 345 | 345 |
|  | **Total** | 1.126.595 | 92 | 14.948 | 6.186 | 5902 | 6535 | 12437 | 1.266.416 | 14 | 4.739 | 3.003 | 901 | 1821 | 2722 | 2.393.011 | 106 | 19.687 | 9.189 | 6803 | 8356 | 15159 |
| **2013** | **1-4** | 74.690 | - | 22 | - | - |  | - | 71.508 | - | 14 | - | - |  | - | 146.198 | - | 37 | - | - | - | - |
|  | **5-9** | 76.258 | - | 79 | 3 | - | 0 | 0 | 72.634 | - | 44 | - | - |  | - | 148.892 | - | 123 | 3 | - | 0 | 0 |
|  | **10-14** | 81.513 | - | 126 | - | - |  | - | 78.255 | 1 | 123 | 3 | 79 | 0 | 79 | 159.768 | 1 | 249 | 3 | 79 | 0 | 79 |
|  | **15-19** | 91.670 | 11 | 1.854 | 748 | 822 | 1.131 | 1.953 | 88.455 | - | 684 | 323 | - | 175 | 175 | 180.125 | 11 | 2.538 | 1.071 | 822 | 1.306 | 2.128 |
|  | **20-24** | 101.822 | 26 | 4.293 | 1.800 | 1.831 | 3.468 | 5.299 | 98.984 | 3 | 1.584 | 918 | 213 | 877 | 1.091 | 200.806 | 29 | 5.876 | 2.718 | 2.045 | 4.345 | 6.390 |
|  | **25-29** | 99.678 | 23 | 3.422 | 1.616 | 1.511 | 1.769 | 3.280 | 101.774 | 1 | 1.223 | 613 | 66 | 336 | 402 | 201.452 | 24 | 4.645 | 2.229 | 1.577 | 2.106 | 3.683 |
|  | **30-34** | 84.075 | 13 | 2.462 | 1.182 | 786 | 1.399 | 2.186 | 94.145 | 3 | 860 | 201 | 181 | 20 | 201 | 178.220 | 16 | 3.322 | 1.383 | 967 | 1.420 | 2.387 |
|  | **35-39** | 70.989 | 6 | 1.307 | 738 | 337 | 729 | 1.066 | 83.858 | 1 | 481 | 157 | 58 | 56 | 113 | 154.847 | 7 | 1.788 | 895 | 395 | 784 | 1.179 |
|  | **40-44** | 73.365 | 10 | 1.487 | 540 | 504 | 560 | 1.065 | 88.168 | 2 | 633 | 111 | 101 | 36 | 137 | 161.533 | 12 | 2.120 | 651 | 605 | 596 | 1.201 |
|  | **45-49** | 88.361 | 7 | 653 | 444 | 310 | 161 | 471 | 106.308 | 1 | 200 | 78 | 48 | 15 | 63 | 194.669 | 8 | 853 | 522 | 357 | 176 | 534 |
|  | **50-54** | 83.741 | 2 | 407 | 292 | 79 | 74 | 153 | 104.483 | - | 98 | 68 | - | 10 | 10 | 188.224 | 2 | 505 | 360 | 79 | 84 | 163 |
|  | **55-59** | 67.428 | 1 | 182 | 149 | 36 | 74 | 111 | 85.982 | - | 48 | 63 | - | 19 | 19 | 153.410 | 1 | 230 | 212 | 36 | 94 | 130 |
|  | **60-64** | 51.597 | 1 | 85 | 105 | 32 | 56 | 88 | 66.467 | - | 21 | 54 | - | 10 | 10 | 118.064 | 1 | 107 | 159 | 32 | 66 | 98 |
|  | **65 y más** | 92.750 | 1 | 75 | 124 | 26 | 122 | 148 | 138.367 | - | 22 | 118 | - | 48 | 48 | 23.117 | 1 | 98 | 242 | 26 | 171 | 197 |
|  | **Total** | 1.137.937 | 102 | 16.455 | 7.741 | 6.275 | 9.545 | 15.820 | 1.279.388 | 12 | 6.036 | 2.707 | 746 | 1.604 | 2.350 | 2.417.325 | 114 | 22.491 | 10.448 | 7.021 | 11.149 | 18.170 |
| **2014** | **1-4** | 74.711 | 1 | 30 | 1 | 91 | 0 | 91 | 71.450 | - | 15 | 1 | - | 0 | 0 | 146.161 | 1 | 46 | 2 | 91 | 0 | 91 |
|  | **5-9** | 76.275 | - | 82 | 2 | - | 1 | 1 | 72.845 | - | 52 | 1 | - | 0 | 0 | 149.120 | - | 135 | 3 | - | 1 | 1 |
|  | **10-14** | 80.375 | 2 | 134 | 13 | 159 | 3 | 162 | 76.907 | - | 94 | 4 | - | 0 | 0 | 157.282 | 2 | 228 | 17 | 159 | 3 | 162 |
|  | **15-19** | 89.877 | 9 | 1.804 | 507 | 674 | 200 | 873 | 86.856 | 5 | 659 | 230 | 379 | 142 | 521 | 176.733 | 14 | 2.463 | 737 | 1.052 | 342 | 1.394 |
|  | **20-24** | 100.149 | 23 | 4.475 | 1.941 | 1.622 | 4.894 | 6.517 | 97.617 | 2 | 1.504 | 852 | 140 | 1.064 | 1.204 | 197.766 | 25 | 5.980 | 2.793 | 1.762 | 5.959 | 7.721 |
|  | **25-29** | 102.310 | 15 | 3.326 | 1.772 | 981 | 2.986 | 3.967 | 102.961 | 1 | 1.164 | 746 | 67 | 631 | 698 | 205.271 | 16 | 4.490 | 2.518 | 1.048 | 3.617 | 4.665 |
|  | **30-34** | 87.303 | 14 | 2.279 | 1.233 | 851 | 1.484 | 2.335 | 96.764 | 5 | 795 | 633 | 306 | 1.076 | 1.382 | 184.067 | 19 | 3.075 | 1.866 | 1.157 | 2.559 | 3.717 |
|  | **35-39** | 74.287 | 6 | 1.266 | 779 | 337 | 1.216 | 1.553 | 87.006 | - | 434 | 372 | - | 236 | 236 | 161.293 | 6 | 1.700 | 1.151 | 337 | 1.452 | 1.789 |
|  | **40-44** | 70.464 | 10 | 1.212 | 459 | 504 | 220 | 724 | 85.236 | 1 | 504 | 281 | 49 | 164 | 213 | 155.700 | 11 | 1.716 | 740 | 553 | 384 | 937 |
|  | **45-49** | 87.144 | 4 | 637 | 483 | 186 | 858 | 1.043 | 104.460 | 3 | 196 | 224 | 142 | 122 | 264 | 191.604 | 7 | 833 | 707 | 328 | 980 | 1.307 |
|  | **50-54** | 85.654 | - | 380 | 304 | - | 191 | 191 | 106.449 | 1 | 113 | 215 | 42 | 115 | 157 | 192.103 | 1 | 494 | 519 | 42 | 307 | 348 |
|  | **55-59** | 70.260 | 1 | 226 | 199 | 34 | 101 | 135 | 90.049 | 2 | 51 | 106 | 71 | 42 | 113 | 160.309 | 3 | 277 | 305 | 105 | 143 | 248 |
|  | **60-64** | 53.538 | 1 | 97 | 92 | 32 | 80 | 112 | 69.131 | - | 31 | 95 | - | 80 | 80 | 122.669 | 1 | 128 | 187 | 32 | 160 | 192 |
|  | **65 y más** | 96.614 | 3 | 69 | 175 | 151 | 57 | 208 | 144.431 | - | 23 | 162 | - | 60 | 60 | 241.045 | 3 | 91 | 337 | 151 | 117 | 268 |
|  | **Total** | 1.148.961 | 90 | 16.017 | 7.960 | 5.622 | 12.289 | 17.911 | 1.292.162 | 20 | 5.636 | 3.922 | 1.195 | 3.733 | 4.929 | 2.441.123 | 110 | 21.653 | 11.882 | 6.817 | 16.023 | 22.840 |
| **2015** | **1-4** | 74.725 | - | 37 | 2 | - | 0 | 0 | 71.380 | - | 13 | 1 | - | 0 | 0 | 146.105 | - | 50 | 3 | - | 1 | 1 |
|  | **5-9** | 76.470 | - | 82 | 2 | - | 0 | 0 | 73.138 | - | 57 | 3 | - | 1 | 1 | 149.608 | - | 138 | 5 | - | 1 | 1 |
|  | **10-14** | 79.239 | 1 | 114 | 3 | 82 | 0 | 82 | 75.745 | - | 86 | 4 | - | 2 | 2 | 154.984 | 1 | 200 | 7 | 82 | 3 | 85 |
|  | **15-19** | 88.248 | 18 | 2.006 | 527 | 1.343 | 425 | 1.768 | 85.288 | 5 | 763 | 241 | 381 | 116 | 497 | 173.536 | 23 | 2.768 | 768 | 1.724 | 541 | 2.265 |
|  | **20-24** | 98.350 | 10 | 4.778 | 2.259 | 704 | 4.421 | 5.125 | 96.145 | 4 | 1.745 | 973 | 285 | 1.071 | 1.356 | 194.495 | 14 | 6.524 | 3.232 | 989 | 5.492 | 6.481 |
|  | **25-29** | 103.893 | 14 | 3.615 | 1.980 | 921 | 3.446 | 4.368 | 103.562 | 1 | 1.430 | 911 | 66 | 1.036 | 1.102 | 207.455 | 15 | 5.045 | 2.891 | 987 | 4.483 | 5.470 |
|  | **30-34** | 90.730 | 9 | 2.304 | 1.451 | 552 | 3.023 | 3.575 | 99.229 | 3 | 897 | 717 | 183 | 606 | 789 | 189.959 | 12 | 3.201 | 2.168 | 736 | 3.629 | 4.365 |
|  | **35-39** | 77.432 | 16 | 1.479 | 919 | 900 | 905 | 1.805 | 90.028 | 1 | 559 | 435 | 54 | 337 | 391 | 167.460 | 17 | 2.037 | 1.354 | 954 | 1.242 | 2.195 |
|  | **40-44** | 69.116 | - | 896 | 604 | - | 559 | 559 | 83.820 | 3 | 312 | 296 | 151 | 234 | 384 | 152.936 | 3 | 1.208 | 900 | 151 | 793 | 944 |
|  | **45-49** | 85.162 | 5 | 1.255 | 471 | 226 | 360 | 587 | 101.975 | - | 567 | 234 | - | 102 | 102 | 187.137 | 5 | 1.822 | 705 | 226 | 463 | 689 |
|  | **50-54** | 86.958 | 3 | 405 | 341 | 120 | 315 | 436 | 107.613 | 1 | 138 | 185 | 42 | 41 | 82 | 194.571 | 4 | 543 | 526 | 162 | 356 | 518 |
|  | **55-59** | 73.033 | - | 268 | 210 | - | 222 | 222 | 93.746 | - | 69 | 141 | - | 81 | 81 | 166.779 | - | 337 | 351 | - | 304 | 304 |
|  | **60-64** | 55.746 | 1 | 106 | 117 | 28 | 67 | 95 | 72.239 | - | 40 | 107 | - | 33 | 33 | 127.985 | 1 | 146 | 224 | 28 | 100 | 129 |
|  | **65 y más** | 100.657 | 1 | 86 | 185 | 24 | 294 | 318 | 150.655 | - | 35 | 180 | - | 113 | 113 | 251.312 | 1 | 121 | 365 | 24 | 408 | 432 |
|  | **Total** | 1.159.759 | 79 | 17.429 | 9.071 | 4.902 | 14.040 | 18.941 | 1.304.563 | 18 | 6.711 | 4.428 | 1.161 | 3.774 | 4.936 | 2.464.322 | 97 | 24.140 | 13.499 | 6.063 | 17.814 | 23.877 |

M *: deaths adjusted for underreporting. L1: lesions in situ. L2: lesions treated.
