# Supplementary material for: Loss of years of healthy life due to road incidents of motorcyclists in the city of Medellin, 2012 to 2015
Source: PLoS One. 2021 Aug 27;16(8):e0256758. doi: 10.1371/journal.pone.0256758 (PMC8396781; doi:10.1371/journal.pone.0256758)
Supplement: S4 Table — (DOCX) [file pone.0256758.s004.docx]

**S4 Table. Distribution of YLD due to motorcyclist road incidents according to the nature of injury and the number of injuries per event.**

| **nature of the injury** | **YLD** | **%** |
| --- | --- | --- |
| **An injury** | **1.810** | **3,4** |
| Severe traumatic brain injury | 741 | 40,9 |
| Contusion anywhere on the body | 199 | 11,0 |
| Femoral neck or hip fracture | 101 | 5,6 |
| Patella, tibia or fibula or ankle fracture | 96 | 5,3 |
| Shoulder dislocation | 93 | 5,1 |
| Radius or ulna fracture | 80 | 4,4 |
| pelvis fracture | 70 | 3,9 |
| Other muscle and tendon injuries (includes sprains, strains and dislocations other than shoulder, knee, hip) | 67 | 3,7 |
| Foot bones fracture | 62 | 3,4 |
| Internal bleeding in the abdomen and pelvis | 51 | 2,8 |
| Crush injury | 44 | 2,4 |
| Spinal cord injury at the neck level | 32 | 1,8 |
| Fracture of the sternum and/or fracture of one or two ribs | 28 | 1,5 |
| Open wound | 27 | 1,5 |
| Facial bone fracture | 21 | 1,1 |
| *Subtotal* | *1711* | *94,5* |
| **Two injuries** | **34.680** | **65,0** |
| Severe traumatic brain injury/ Contusion to any part of the body | 7.096 | 20,5 |
| Severe traumatic brain injury/ Open wound | 2.112 | 6,1 |
| Patella, tibia or fibula or ankle fracture / Contusion anywhere on the body | 1.854 | 5,3 |
| Severe traumatic brain injury/ Other muscle and tendon injuries (includes sprains, strains and dislocations other than shoulder, knee, hip) | 1.809 | 5,2 |
| Pelvis fracture  / Contusion anywhere on the body | 1.645 | 4,7 |
| Crush injury/ Contusion anywhere on the body | 1.385 | 4,0 |
| Radius or ulna fracture / Contusion anywhere on the body | 1.158 | 3,3 |
| Foot bones fracture / Contusion anywhere on the body | 1.031 | 3,0 |
| Internal bleeding in the abdomen and pelvis/ Contusion anywhere on the body | 992 | 2,9 |
| Other muscle and tendon injuries (includes sprains, strains and dislocations other than shoulder, knee, hip)/ Contusion anywhere on the body | 953 | 2,7 |
| Shoulder dislocation/ Contusion anywhere on the body | 876 | 2,5 |
| Contusion anywhere on the body / Open wound | 716 | 2,1 |
| Spinal cord injury at the neck level/ Contusion anywhere on the body | 653 | 1,9 |
| Facial bone fracture / Contusion anywhere on the body | 633 | 1,8 |
| Moderate traumatic brain injury / Contusion anywhere on the body | 508 | 1,5 |
| *Subtotal* | *23421* | *67,5* |
| **Three injuries** | **15.403** | **28,9** |
| Severe traumatic brain injury / Contusion anywhere on the body/ Open wound | 2.769 | 18,0 |
| Severe traumatic brain injury / Other muscle and tendon injuries (includes sprains, strains and dislocations other than shoulder, knee, hip)/ Contusion anywhere on the body | 788 | 5,1 |
| Patella, tibia or fibula or ankle fracture / Other muscle and tendon injuries (includes sprains, strains and dislocations other than shoulder, knee, hip)/ Contusion anywhere on the body | 772 | 5,0 |
| Internal bleeding in the abdomen and pelvis/ Facial bone fracture/ Contusion anywhere on the body | 492 | 3,2 |
| Severe traumatic brain injury / Eye injury/ Contusion anywhere on the body | 460 | 3,0 |
| Other muscle and tendon injuries (includes sprains, strains and dislocations other than shoulder, knee, hip)/ Contusion anywhere on the body/ Open wound (a corto plazo, con o sin tratamiento) | 437 | 2,8 |
| Severe traumatic brain injury / Other muscle and tendon injuries (includes sprains, strains and dislocations other than shoulder, knee, hip)/ Open wound (short-term, with or without treatment) | 373 | 2,4 |
| Radius or ulna fracture/ Other muscle and tendon injuries (includes sprains, strains and dislocations other than shoulder, knee, hip)/ Contusion anywhere on the body/ Open wound | 354 | 2,3 |
| Foot bones fracture/ Other muscle and tendon injuries (includes sprains, strains and dislocations other than shoulder, knee, hip)/ Contusion anywhere on the body/ Open wound | 349 | 2,3 |
| Patella, tibia or fibula or ankle fracture / Foot bones fracture/ Contusion anywhere on the body | 344 | 2,2 |
| Severe traumatic brain injury / Facial bone fracture/ Open wound | 320 | 2,1 |
| Severe traumatic brain injury / Foot bones fracture/ Contusion anywhere on the body | 316 | 2,1 |
| Moderate traumatic brain injury / Radius or ulna fracture/ Contusion anywhere on the body | 290 | 1,9 |
| Severe traumatic brain injury / Eye injury/ Open wound | 277 | 1,8 |
| Patella, tibia or fibula or ankle fracture / Contusion anywhere on the body/ Open wound | 276 | 1,8 |
| *Subtotal* | *8619* | *56,0* |
| **Four injuries** | **1.448** | **2,7** |
| Severe traumatic brain injury / Hand fracture / Contusion anywhere on the body/ Open wound | 331 | 22,9 |
| Severe traumatic brain injury / Facial bone fracture/ Contusion anywhere on the body/ Open wound | 314 | 21,7 |
| Facial bone fracture/ Radius or ulna fracture/ Other muscle and tendon injuries (includes sprains, strains and dislocations other than shoulder, knee, hip)/ Contusion anywhere on the body | 201 | 13,9 |
| Severe traumatic brain injury / Crush injury/ Facial bone fracture/Other muscle and tendon injuries (includes sprains, strains and dislocations other than shoulder, knee, hip) | 172 | 11,9 |
| Severe traumatic brain injury / Skull fracture/ Radius or ulna fracture/ Hand fracture | 82 | 5,7 |
| Fracture of the sternum and/or fracture of one or two ribs/ Radius or ulna fracture/ Foot bones fracture/ Contusion anywhere on the body | 73 | 5,1 |
| Shoulder dislocation/ Other muscle and tendon injuries (includes sprains, strains and dislocations other than shoulder, knee, hip)/ Contusion anywhere on the body/ Open wound | 63 | 4,4 |
| Moderate traumatic brain injury / Facial bone fracture/ Contusion anywhere on the body/ Open wound | 48 | 3,3 |
| Severe traumatic brain injury / Eye injury/ Other muscle and tendon injuries (includes sprains, strains and dislocations other than shoulder, knee, hip)/ Open wound | 39 | 2,7 |
| Patella, tibia or fibula or ankle fracture / Foot bones fracture/ Other muscle and tendon injuries (includes sprains, strains and dislocations other than shoulder, knee, hip)/ Contusion anywhere on the body | 38 | 2,6 |
| Patella, tibia or fibula or ankle fracture / Foot bones fracture/ Contusion anywhere on the body/ Open wound | 34 | 2,3 |
| Facial bone fracture/ Patella, tibia or fibula or ankle fracture / Foot bones fracture/Other muscle and tendon injuries (includes sprains, strains and dislocations other than shoulder, knee, hip) | 27 | 1,9 |
| Moderate traumatic brain injury / Facial bone fracture/ Other muscle and tendon injuries (includes sprains, strains and dislocations other than shoulder, knee, hip)/ Open wound | 26 | 1,8 |
| Subtotal | *1.448* | *100,0* |
| **Total** | **53.342** | **100,0** |
