# Supplementary material for: Loss of years of healthy life due to road incidents of motorcyclists in the city of Medellin, 2012 to 2015
Source: PLoS One. 2021 Aug 27;16(8):e0256758. doi: 10.1371/journal.pone.0256758 (PMC8396781; doi:10.1371/journal.pone.0256758)
Supplement: Attachment — Submitted filename: Response to Reviewers.docx [file pone.0256758.s008.docx]

| **Reviewers' comments** | **Response to Reviewers** |
| --- | --- |
| **Please include the following items when submitting your revised manuscript:**  A rebuttal letter that responds to each point raised by the academic editor and reviewer(s). You should upload this letter as a separate file labeled 'Response to Reviewers'.  A marked-up copy of your manuscript that highlights changes made to the original version. You should upload this as a separate file labeled 'Revised Manuscript with Track Changes'.  An unmarked version of your revised paper without tracked changes. You should upload this as a separate file labeled 'Manuscript'. | We have included the requested elements in this resubmission of the manuscript. |
| If you would like to make changes to your financial disclosure, please include your updated statement in your cover letter. Guidelines for resubmitting your figure files are available below the reviewer comments at the end of this letter | We have made changes to the financial disclosure which is indicated on the cover letter. |
| **Journal Requirements** |  |
| Please ensure that your manuscript meets PLOS ONE's style requirements, including those for file naming. The PLOS ONE style templates can be found at: <https://journals.plos.org/plosone/s/file?id=wjVg/PLOSOne_forma>  [tting_sample_main_body.pdf](https://journals.plos.org/plosone/s/file?id=wjVg/PLOSOne_formatting_sample_main_body.pdf) and https://journals.plos.org/plosone/s/file? id=ba62/PLOSOne_formatting_sample_title_authors_affiliations.pdf | We have ensured that the manuscript meets the style requirements of the PlosOne journal. |
| We note that you have included text in the title page that is not in English, as per the journal guidelines (<https://journals.plos.org/plosone/s/submission-guidelines>) manuscripts must be submitted in English, please therefore update the title page text to English. | We have made the correction, which is located between lines 14 and 19 |
| We note you have included a table to which you do not refer in the text of your manuscript. Please ensure that you refer to Table 1 in your text; if accepted, production will need this reference to link the reader to the Table. | This Table 1 reference is in lines 264, 266 and 279 |
| Please include captions for your Supporting Information files at the end of your manuscript, and update any in-text citations to match accordingly. Please see our Supporting Information guidelines for more information: <http://journals.plos.org/plosone/s/supporting-information> | Information file legends were included at the end of the manuscript and in-text citations were updated. |
| **Review Comments to the Author**  **General comments** | |
| **Reviewer #1** | |
| **Comment** | **Response to reviewer** |
| 1. I am missing explicit information on the exact purpose of the study | Detailed information on the objective of the study can be found between lines 100 and 113. |
| 2. Please, include as much information as possible about the population/sample of the study, cases analyzed, sociodemographic. It would be of great help to have the materials and methods sections divided into subsections. | Information related to motorcyclist fatalities and injuries from road incidents was appended to the manuscript, and the materials and methods section was further divided into subsections. |
| 3. I recommend the authors include a “limitations and further studies” (or similar) section in which the potential analyses not performed in this study (inferential comparisons, prediction) and other matters could be addressed | Indeed, we have included a section called “limitations and further studies”, which can be seen between lines 445 and 587. |
| 4. Also, I would find it interesting and useful as a reader to have a “practical implications” section | We have included a section entitled "practical implications", which can be seen between lines 550 and 583. |
| 5. few format issues should be addressed (e.g., some paragraphs without indentation as in line 236, line spacing in paragraph 176). One could more comfortably read the article with a homogeneous format. Please, review the general format of the paper including the tables | We corrected the text. |
| **Specific comments:** | |
| **Comment** | **Response to reviewer** |
| 6. I encourage the authors to rewrite the first sentence (lines 46-47). If the authors want to highlight this terminological clarification as in the reference provided, I believe further explanation could be given to ease the readers with the understanding of the matter of study. Maybe there is a conceptualization misunderstanding between road injuries and road crashes (see <https://doi.org/10.18270/cuaderlam.v16i30.2842>). A car may be involved in a traffic crash but injured is not a term usable for cars, but persons instead. The use given to the term road injuries (of Motorcyclists) in the title would be appropriate. Please, review it throughout the paper. | We clarified the conceptualization between traffic injuries and traffic accidents to facilitate the understanding of the subject of the study, which can be found between lines 45 and 58. The adjustment was made throughout the document. |
| 7. Line 52, I would give this source of information as a reference (instead of as text) to make it easier for the reader to access the data. | We corrected the text. |
| 8. Line 65, GDP, when abbreviations are used for the first time the full meaning of the abbreviation should be given. Also, ICD-10. | We corrected the text. |
| 9. There are several lines (e.g., 221, 241, 301) where “Error! Reference source not found” is written, correct this. | We corrected the text. |
| 10. The references section should be thorough reviewed. There are a considerable number of format and citing errors that must be addressed. Please follow the referencing style guidelines of the journal. | We checked all references and made the necessary corrections. |
| 10.1 Please, do not use both the Spanish and English acronym for World Health Organization. | We made the correction throughout the text |
| 10.2 Please, translate Spanish terms into English where appropriate as required by normative. | We made the correction throughout the text |
| 10.3 Do not cite journal articles as webpages | Since there are references that do not have DOI or PMID, we have chosen to place the URL of the article being mentioned |
| 10.4 Do not include the term “volume” before the number of the volume. | We have made the adjustment in the references |
| 10.5 The month and day of publications are not required. | We have made the adjustment in the references |
| **Reviewer #2** | |
| **Comment** | **Response to reviewer** |
| This interesting study aims to estimate disease burden in terms of disability-adjusted life years (DALYs) lost due to RIs of motorcyclists in the city of 96 Medellín between 2012 and 2015. The information sources were the Single Register of Affiliates, death certificates from the vital record office (RUAF-D), the database of injured persons according to the Police reports of traffic accidents at the scene ‒“in situ”‒, provided by the Mobility Secretariat of Medellín and the Individual Registries of Provision of Services (RIPS) of the outpatient, hospitalization, and emergency services. Of the total number of RIPS of the outpatient, hospitalization, and emergency services, a selection was made which included the records containing (as a diagnosis—main or related) an ICD-10 code associated at least with one of the injuries considered [11]; The analysis of YLL (using average life expectancy values) and YLD for single injuries followed standard global burden of disease estimates however the YLD adjustment for multiple injuries sustained by the same patient appears novel. According to death registrations and police reports there were 428 deaths over the study period, 80% in men and 87,971 injury incidents.  The DALYs analysis, a rate of 80,000 DALYS per 100,000 over the period of study is not directly comparable to the WHO estimate of 319 per 100,000 for Motorcycle incidents in Colombia in 2019. However, as the latter has a lifetime horizon for YLD it would be expected to be higher. This does call into question the methods used in this study as the DALYS annual rate is per year 60 times greater than the WHO estimate, and also suggests that most of the disease burden came from non-fatal injuries which runs contrary to GBD studies. The findings are interesting however the following clarifications are needed. | We believe there was a misunderstanding. 80,046 is not a rate; it is an absolute number that indicates the number of disability-adjusted life years (DALYs). Regarding the doubts generated by the differences between our estimate and that of the WHO, we have the following argumentation (between lines 380 and 387, 388 and 400 and 430 and 443):  During the four years of study, the loss of healthy life years due to motorcyclist road incidents in Medellin was 80,046 DALYs (in absolute terms), which represented a rate of 823.8 DALYs per 100,000 inhabitants. When compared to Colombia’s rate for 219 according to WHO, 319 per 100,000, the annual rate of disability-adjusted life years (DALYs) is only 2.6 times higher than the WHO estimate. To consider these differences, it should be kept in mind that the studies conducted worldwide on burden disease are based on predictive models that include meta-analysis and Bayesian models.  In Colombia, it could yield estimates of the burden disease due to road incidents involving motorcyclists debatable due to the lack of a culture of information and consequently difficulties in accessing reliable sources of information and good coverage. In the case of Medellin, however, it was possible to estimate the epidemiological parameter by nature of injury associated with motorcyclist road incidents through secondary information sources generated from vital statistics and health care records; therefore, it was not necessary to develop prediction processes, which would provide greater reliability to the results obtained. Furthermore, unlike other studies that have been carried out outside the country, in Medellin, we considered the injuries of motorcyclists treated in the outpatient service and not only those admitted to hospitalization and emergency services, with a higher level of severity. |
| Data on about 3,000 patients has been made available in the submission but there is no statement on where the other data sources for mortality and incidents can be accessed | The sources of information used are included in the manuscript (S1 Datasets.xls, S2 Datasets.xls, S3 Datasets.xls, S1 Table.docx, S2 Table.docx, S3 Table.docx, S4 Table.docx) |
| **Methods:** | |
| Please clarify if and how the police reports were linked to hospital data to ensure accurate estimates of non-fatal injury and address the data availability queries above | Police reports were not linked to data hospital due to the impossibility of having an identifier that would allow applying a relationship identity model. |
| **Results** |  |
| Produce a further descriptive table of the 87,971 incidents detailing how many resulted in hospital admission, non-presentation to healthcare, or outpatient attendance only cross tabulating frequencies of injuries sustained in those killed or admitted – TBI, spinal injury, thoraco abdominal injury, limb injury. | The data on non-fatal injuries were processed from two independent sources of information, so it is not possible to prepare the table in the requested format; however, the number of health care services, some characteristics of this unit of analysis, the disaggregation by health service (outpatient, hospitalization and emergency) and by type of injury are presented (between lines 266 and 269, 283 and 296).  Given the limitations of the information, it was impossible to know the distribution of non-visits with a doctor or outpatient care. |
| **Discussion:** | |
| Defend the use of the novel calculation of YLD for multiply injured patients, comment further on the large discrepancy between these estimates and those of WHO in 2019. | An adjustment according to the multiplicative model was necessary due to the fact that the YLDs could be overestimated if a significant volume of individuals presented multiple injuries due to the same incident. Furthermore, if the loss of health for each injury were to be counted separately, it would reflect considerably inflated results.  Because about 7% of the events attended in the health services presented more than two natures of injury generated by the same road incident, the calculation of the DALYs was adjusted through a multiplicative model, which allowed this indicator to reflect the sum of the total health lost in the individual. Thus, individuals with the same loss of functional health were then treated as if they were equal, regardless of whether that loss of functional health stemmed from one or more contributing conditions (between lines 417 and 422, 218 and 222). |
| Comment on the use of helmets by motorcyclists in Colombia for context | Despite the existence of legislation in Colombia related to the mandatory use of motorcycle helmets as elements of road safety for the population, problems have been encountered in its compliance, first for climatic, aesthetic and hygienic reasons, and second, because of the perception of risk and the possibility of being stopped and sanctioned by the authorities is increasingly lower. In Colombia, after the year 2012, a regulation for the use of helmets was implemented, which emphasizes the mandatory use of helmets and determines punitive measures. However, in a qualitative study in Valledupar, Colombia, it was identified that the helmet is not internalized in the population as an object that provides safety and protects life; it is used to avoid a traffic ticket, being used intermittently and inappropriately. Moreover, its use is differential by geographic regions, where social and economic development is key factors (between lines 567 and 577).  Thus, the call is for everyone to commit themselves to take care of their own lives and the lives of others through their actions and responsibilities |

**HUGO GRISALES ROMERO	SANDRA MILENA PORRAS CATAÑO**
