# Supplementary material for: Loss of years of healthy life due to road incidents of motorcyclists in the city of Medellin, 2012 to 2015
Source: PLoS One. 2021 Aug 27;16(8):e0256758. doi: 10.1371/journal.pone.0256758 (PMC8396781; doi:10.1371/journal.pone.0256758)
Supplement: Attachment — Submitted filename: Response to reviewer and editor_Version_2.docx [file pone.0256758.s009.docx]

| **Reviewer #1's comments** | **Responses to reviewer** | |
| --- | --- | --- |
| **Please include the following items when submitting your revised manuscript:**  A rebuttal letter that responds to each point raised by the academic editor and reviewer(s). You should upload this letter as a separate file labeled 'Response to Reviewers'.  A marked-up copy of your manuscript that highlights changes made to the original version. You should upload this as a separate file labeled 'Revised Manuscript with Track Changes'.  An unmarked version of your revised paper without tracked changes. You should upload this as a separate file labeled 'Manuscript'. | We have included the requested elements in this resubmission of the manuscript. | |
| Please, add a reference in the first paragraph. | We have included a reference in the first paragraph (Line 50) | |
| Lines 46-48, consider rewriting the sentence from “In this phenomenon” to “combined.”. | We corrected the text (Lines 46-47). | |
| Line 121-123, consider rewriting. | We rewrote the paragraph. This is the improved version: “These were all records of deaths and injuries due from RIs of motorcyclists, collected from different sources, in the city of Medellin between 2012 and 2015” (Lines 133-134). | |
| Line 257, consider adding the percentage for unmarried cases. | We have added the percentage for unmarried cases.  It is 53.7% (Line 268). | |
| Line 261 and 290, add .0 to the 82% and 98% | We corrected the text (Line 272 and Lines 301-303) . | |
| Line 272, IQR should be only a number as it presents de distance between quartiles 25th and 75th. If the authors are expressing the quartiles 25 and 75, please correct it. | We corrected the text (Line 283). | |
| Line 291, I am not sure what this 25% inside the parenthesis stands for. | We corrected the text: “When asked about the type of affiliation to the social security system in Colombia, it was found that 25.5% were affiliated to the contributory system (n: 11,449)” (Lines 301-303) | |
| Line 437 and 440, Consider adding a full stop instead of a semicolon before “in consistency” and “therefore”, respectively. | We corrected the text. (Line 451 and Line 454) | |
| References, I encourage the authors to carefully check all the references. | We have carefully reviewed the references and made the necessary adjustments. | |
| I would remove the PMID if already giving the doi. | We made the requested adjustments (References 25 (Line 769) and 26 (Line 773)) | |
| Reference 36, Injury Prevention is the name of the journal I am not sure what does 6.4 stands for, please check it and correct it if necessary. | We corrected the text (Reference 40 (Line 824)) | |
| Reference 39, “Ciênc. saúde colet” should be each word capitalized as it is the name of the journal. Same for reference 28. | We made the requested adjustments (Line 836 and Line 789) | |
| **Academic editor review** | | |
| Personally, and after a careful read of your revised paper, I would also like to ask you to strengthen the literature review, specifically in regards to the empirical evidences on human factors and road safety, e.g., risky road behaviors in the context of Medellín, or at least Colombian/Latin American cities, and the relationship between the specific road environment of the country and toad behaviors and crashes. This is especially problematic if the fact that most of the papers empirically documenting this issue were not considered in your manuscript, and many of the study assumptions were done on the basis of non-scientific sources. In other words, in its present form, the paper lacks from a clear scientific support on the road safety panorama of Colombian drivers (including motorcyclists). | | |
| **Response to academic editor** | | |
| We consider that our study has a very clear aim, which is the estimation of the burden of disease due to road incidents of motorcyclists, and does not focus on the factors that have an impact on its occurrence, such as the human factor. However, we decided to accept your suggestion, given the importance of the relationship between the human factor and road safety, and therefore we annexed to the manuscript, in some sections, especially in the one related to practical implications, aspects that relate the human factor and road safety. These are indicated below (the line numbers serve as an orientation for the immediate location of the text in the manuscript with no change control): | | |
| In this regard, other factors that have been related to the higher death rates due to RIs, in general, in drivers under 25 years of age, are the lack of experience, unsafe attitudes, sensation seeking, lower risk perception and lower use of safety measures^[1]^ .  There are other endogenous factors to be taken into account in RIs, especially the human factor. In Colombia, Norza et al. in 2014, in a sample of 16,322 drivers, reported the role of motorcyclists in road incidents, probably attributed to the disregard of traffic regulations, which added the fact of the lack experience behind the wheel that characterizes these people. The long working hours combined with the drivers' lack of emotional intelligence could be triggers of stress, anxiety, anger, aggressiveness, hostility, dissociative disorders, and consequently, factors that facilitate the tendency to commit more infractions and RIs ^[2]^ . Also, in three Colombian cities, Ibague-Valledupar, and Bogotá, two studies ratified the precept of the multi-causality of traffic accidents, where human factors are the ones that most contributed to the occurrence of incidents, aggravated by apparent deficiencies in infrastructure and some organizational problems^[3],^^[4]^.  The combination of three factors, human, environmental, and automotive, constitute the fundamental elements to be considered in road events. In particular, knowledge of the human factor is essential in road safety to guide intervention strategies and prevent accidents and their causes. The human factor is the most difficult to control and the most influential because driving skills and habits are decisive for accident prevention. Although in theory, drivers, in general, should be in optimal psychophysical conditions when driving, in practice, this is not usual, and therefore deficiencies in psychophysical health can trigger events that can life-threatening to drivers, passengers, and pedestrians. In particular, studies addressing the human factor analysis in road incidents involving motorcyclists are scarce, probably due to the complexity of the evaluation of human characteristics.    Some studies have focused on the human factor in motorcycle drivers involved in RIs in Latin American countries. A descriptive study in Paraguay for this specific population concluded that the greatest attribution to the occurrence of road events was due to behavioral factors^[5]^ . In Argentina, the costs and factors derived from motorcycle accidents were analyzed, highlighting that the human factor was the leading cause, especially the lack of respect for traffic regulations, within which speeding when performing maneuvers can cause harm to the driver and passengers ^[6]^ . In Caracas, in a case-control design, a positive association was found between the presence of Attention Deficit Hyperactivity Disorder (ADHD) and a higher occurrence RIs of motorcyclist in people between 18 and 63 years of age, i.e., motorcycle drivers who presented the disease had a higher risk of experiencing RIs incidents due to their greater inclination to violate traffic laws and to have inappropriate behaviors than motorcycle drivers who did not present ADHD^[7]^.  In Medellin in 2015^[8]^, the psychological profile of drivers who repeatedly violated traffic rules were analyzed, identifying that reckless, risky, and aggressive driving, disrespect for traffic rules, emotional instability, especially anxiety in young drivers, and dissociative personality were related to attentional errors in driving and traffic incidents. The greater impact on road events in the young population could be explained by their perception of risk, which is also affected by perceptual abilities, driving experience, emotional state, and traffic regulations^[9]^ .  In particular, the trait of road rage, as a human factor, has been little studied in the context of professional drivers. Useche et al., in 2019^[10]^, analyzed the association between trait road rage and driving styles in a sample of Colombian professional drivers, highlighting, among the main findings that impeding forward progress by other drivers, illegal driving and hostility significantly predicted adaptive and maladaptive driving styles. It was concluded that interventions that focus on minimizing the driving anger profile of drivers could be effective in counteracting the consequences of such predisposition. Apart from the behavioral problems referred to above, motorcycle drivers in the city of Medellin are known for their special predisposition to contravene road regulations. In a comparative study on the profile of motorcyclists in four Colombian cities, Bogotá, Medellin, Cucuta, and Bucaramanga^[11]^ , it was found that it is in Medellin where this type of driver violates the National Traffic Code to a greater extent, and it was concluded that the motorcyclist‘s profile is different depending on the region they are from and therefore it is ratified that generalized public policies cannot be carried out by local governments towards issues related to motorcycles.  The human factor is the most difficult to control and is influential since driving skills and habits determine accident prevention. In this sense, behavioral interventions are essential for structuring safe road systems and, therefore, require the design of intervention programs to change driver behavior^[12]^. Necessarily, in the perspective of minimizing the risks inherent to the human factor, prior knowledge of the causal factors is required through antecedent and consequent communication strategies with the driver and the application of principles of social influence, which allow creating suitable conditions for the desired behavior to occur. | | Lines 462-465  Lines 66-77  Lines 594-604  Lines 605-618  Lines 619-625  Lines 627-642  Lines 643-650 |

**HUGO GRISALES ROMERO	SANDRA MILENA PORRAS CATAÑO**

1. Useche S. A., Alonso F, Montoro, L, Esteban C. Explaining self-reported traffic crashes of cyclists: An empirical study based on age and road risky behaviors. Safety science. 2019; 113: 105-114. doi: [https://doi.org/10.1016/j.ssci.2018.11.021](https://doi.org/10.1016/j.ssci.2018.11.021)
2. Norza-Céspedes EH, Granados-León EL, Useche-Hernández SA, Romero-Hernández M, Moreno-Rodríguez J. Descriptive and explanatory components of road accident rate in Colombia: influence of the human factor. Revista criminalidad. 2014; 56(1):157-187.Available: <http://www.scielo.org.co/scielo.php?script=sci_arttext&pid=S1794-31082014000100009>
3. Flórez CF, Patiño C, Rodríguez JM, Ariza LK, LIANY; González RA. Análisis multicausal de ‘accidentes’ de tránsito en dos ciudades de Colombia. Archivos de Medicina (Manizales). 2018; 18(1); 69-85. doi:: <https://doi.org/10.30554/archmed.18.1.2477.2018>
4. Castellanos OJ. Análisis de la tasa de accidentalidad de motocicletas entre los años 2005–2017 en la ciudad de Bogotá DC.Universidad Cooperativa de Colombia. 2018. Available: https://repository.ucc.edu.co/bitstream/20.500.12494/10185/1/2018_%20Analisis_Accidentalidad_Motocicletas.pdf
5. Negret MM, Sequera G. Factores sociales, ambientales y comportamentales relacionados con los siniestros de tránsito que involucran motocicletas en Asunción, Paraguay. Revista Científica OMNES. 2020; 3(2): 79-109.  Available: https://www.columbia.edu.py/investigacion/ojs/index.php/OMNESUCPY/article/view/72
6. Besse M, Denari R, Villani A, San Roque M, Rosado J, Sarotto AJ. Accidentes de moto: costo médico/económico en un hospital municipal de la ciudad de Buenos Aires. MEDICINA (Buenos Aires). 2018; 78(3):158-162. Available: https://www.medicinabuenosaires.com/revistas/vol78-18/n3/158-162-Med6767-Besse.pdf
7. Suárez I, Rosales-Pereira KA, Silva AA, González G, López K, Rísquez A. Déficit de Atención e Hiperactividad. Su relación con Accidentes de Tránsito en Motocicletas. Medicina Interna. 2019; 35(3): 99-106. Available:  https://www.svmi.web.ve/ojs/index.php/medint/article/view/520
8. Durán-Palacio NM, Moreno-Carmona ND. Personalidad e infracciones frecuentes de normas de tránsito. Diversitas. 2016; 12(1): 123-136. doi: <https://doi.org/10.15332/s1794-9998.2016.0001.09>
9. Payani S, Hamid H, Law TH. A review on impact of human factors on road safety with special focus on hazard perception and risk-taking among young drivers. In IOP Conference Series: Earth and Environmental Science. 2019; 357(1). Available: https://iopscience.iop.org/article/10.1088/1755-1315/357/1/012041/meta.
10. Useche SA, Cendales B, Alonso F, Montoro L, Pastor JC. Trait driving anger and driving styles among Colombian professional drivers. Heliyon. 2019); 5(8). doi: <https://doi.org/10.1016/j.heliyon.2019.e02259>
11. Martin-Rojas DA, Pardo-Castaño, D. Estudio comparativo del perfil del motociclista en cuatro ciudades de Colombia: Bogotá, Medellín, Cúcuta y Bucaramanga. Ciencia Unisalle. 2019. Available: https://ciencia.lasalle.edu.co/ing_civil/535
12. Theeuwes J, van der Horst R, Kuiken M. Designing safe road systems : a human factors perspective. Jan Series: Human factors in road and rail transport Publisher: CRC Press. 2017
